# A Highly Efficient Environmental-Friendly Adsorbent Based on Schiff Base for Removal of Cu(II) from Aqueous Solutions: A Combined Experimental and Theoretical Study

**DOI:** 10.3390/molecules26175164

**Published:** 2021-08-26

**Authors:** Said Tighadouini, Othmane Roby, Smaail Radi, Zouhair Lakbaibi, Rafik Saddik, Yahia N. Mabkhot, Zainab M. Almarhoon, Yann Garcia

**Affiliations:** 1 Laboratory of Organic Synthesis, Extraction and Valorization, Faculty of Sciences Ain Chock, Hassan II University, BP 5366, Casablanca 20000, Morocco; othmaneroby1@gmail.com (O.R.); rafik.saddik@gmail.com (R.S.); 2 LCAE, Faculty of Sciences, University Mohamed Premier, Oujda 60000, Morocco; 3 Laboratory of Molecular Chemistry, Materials and Environment (LCME2), Multidisciplinary Faculty Nador, University Mohamed Premier, Oujda, BP 300 Selouane, Nador 62700, Morocco; lakbaibi.zouhair@gmail.com; 4 Department of Pharmaceutical Chemistry, College of Pharmacy, King Khalid University, P.O. Box 960, Abha 61421, Saudi Arabia; 5 Department of Chemistry, College of Science, King Saud University, P.O. Box 2455, Riyadh 11451, Saudi Arabia; zalmarhoon@ksu.edu.sa; 6 Institute of Condensed Matter and Nanosciences, Molecular Chemistry, Materials and Catalysis (IMCN/MOST), Université Catholique de Louvain, Place Louis Pasteur 1, 1348 Louvain-la-Neuve, Belgium

**Keywords:** heavy metal pollution, environment, hybrid materials, water cleaning technologies, DFT

## Abstract

Removal of heavy metals from drinking water sources and rivers is of strategic health importance and is essential for sustainable ecosystem development, in particular in polluted areas around the globe. In this work, new hybrid inorganic-organic material adsorbents made of ortho- (**Si-o-OR**) or para-Schiff base silica (**Si-p-OR**) were synthesized and characterized in depth. These hybrid adsorbents show a high selectivity to Cu(II), even in the presence of competing heavy metals (Zn(II), Cd(II), and Pb(II)), and also demonstrate great reusability after five adsorption-desorption cycles. Maximum sorption capacity for Cu(II) was found for **Si-o-OR** (79.36 mg g^−1^) and **Si-p-OR** (36.20 mg g^−1^) in no less than 25 min. Energy dispersive X-ray fluorescence and Fourier transform-infrared spectroscopy studies demonstrate that this uptake occurs due to a chelating effect, which allows these adsorbents to trap Cu(II) ions on their surfaces; this result is supported by a theoretical study for **Si-o-OR**. The new adsorbents were tested against real water samples extracted from two rivers from the Oriental region of Morocco.

## 1. Introduction

In recent years, industrial sites contaminated by heavy metals have contributed to growing pollution of the overall ecosystem [1]. Heavy metals are not biodegradable, and therefore tend to accumulate and remain in water bodies for a long time. This leads to long-term detrimental effects to the ecosystem and human health in general. Ref. [2] Amongst these toxic metals, Cu(II) ions, as well as others, accumulate in the body even when present at quite a low concentration, resulting in health problems and leading to an irritation of the central nervous system, stomach discomfort, decreased kidney function, mucosal irritation and even development of Alzheimer’s disease, to name but a few [3].

A suitable method is thus needed for removing and selectively recovering Cu(II) from drinkable water. Currently, several remediation approaches and techniques have been applied to capture Cu(II) ions, such as ion exchange [4], coagulation [5], liquid-phase extraction [6], membrane separation [7], electrodeposition [8] and reverse osmosis [9].

Adsorption is actually considered a privileged method due to its economic simplicity, environmental friendliness and low energy requirements [10]. However, the first adsorbents were found to display low removal efficiency as well as low regeneration capability; furthermore, they do not possess selective adsorption capacity in the presence of a mixture of metal ions [11]. Recent research has focused on mesoporous silica due to its high specific surface area, absence of swelling, and fast kinetics, as well as their thermal, mechanical stabilities and compatibilities with other materials [12,13,14]. Recently, chemical engineering of organically modified porous silica has attracted attention for the adsorption of metal ions in environmental remediation [15,16,17,18,19]. Their adsorption performances mainly depend on donor atoms within the incorporated organic moieties, which can form chelating structures with metal ions, e.g., Schiff bases with Cu(II) [20,21,22,23,24].

Schiff base-functionalized siloxanes have been discovered as promising candidates for decreasing toxic metal concentrations in water [25,26]. On the other hand, these materials and their complexes have also generated an interest in homogeneous catalysis [27,28].

In the present work, we have designed new adsorbents via the covalent immobilization of two furan derivatives on silica particles, namely **Si-o-OR** and **Si-p-OR**. The effect of contact time, pH, initial metal ion concentration temperature, recyclability, and coexisting metal ions on the adsorption of Cu(II), Zn(II), Cd(II) and Pb(II) was explored. The adsorption mechanism was investigated through FTIR spectroscopy and computational means. A strong coordination of Cu(II) in a monodentate chelating fashion with a nitrogen atom of the azomethine group of the two studied Schiff bases was evidenced, with superior characteristics demonstrated by **Si-o-OR**. The selected adsorbent was used to successfully remove Cu(II) from aqueous solutions, including real river water samples, with a high maximum sorption capacity of 79.36 mg g^−1^.

## 2. Results

### 2.1. Linker Synthesis

**Scheme 1:**
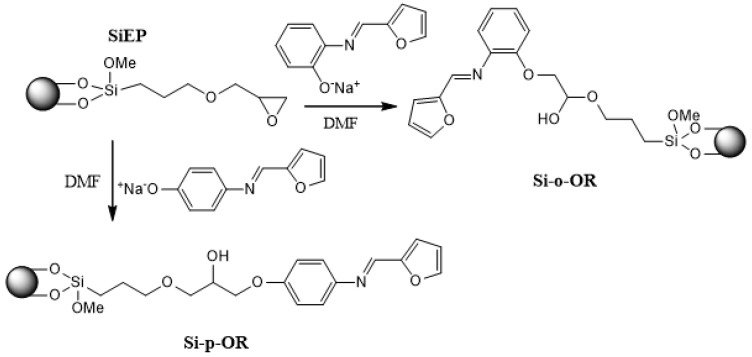
Synthesis route of **Si-p-OR** and **Si-o-OR**.

The synthesis route of the new hybrid materials is outlined in Scheme 1.

The first stage concerns the target ligands, (E)-4-(furan-2-ylmethyleneamino) phenol or (E)-2-(furan-2-ylmethyleneamino) phenol. The next step involves condensation of activated silica gel with 3-glycidoxypropyltrimethoxysilane in refluxing toluene, which afforded epoxy groups on the silica surface **SiEp**. Finally, the epoxy groups grafted on the silica surface were then reacted to yield new hybrid materials named ortho-Schiff base silica **Si-o-OR** and para-Schiff base silica **Si-p-OR**.

### 2.2. Characterization of **Si-p-OR** and **Si-o-OR**

Chemical analysis was as shown herein in (%): C, 6.38; H, 1.32; and N, 1.04 for **Si-o-OR**; and C, 5.84; H, 1.34; and N, 1.13 for **Si-p-OR**. This allowed for the confirmation of the successful syntheses of both materials.

**Figure 1:**
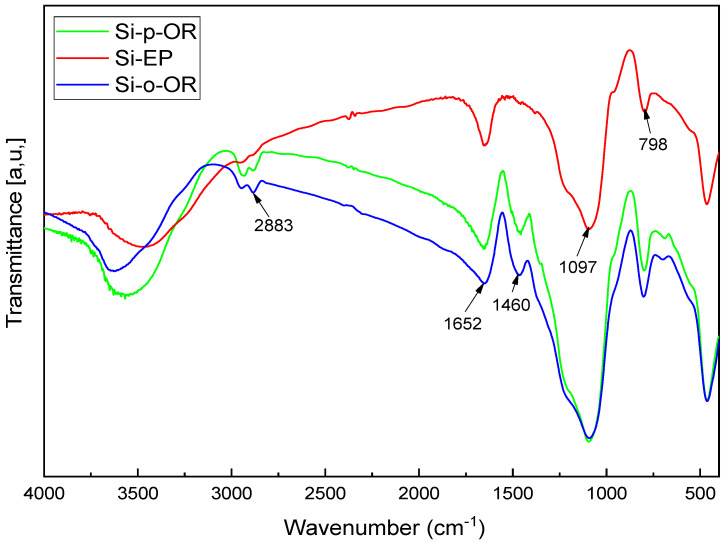
FTIR spectra of **Si-EP**, **Si-o-OR** and **Si-p-OR**.

The FTIR spectra of **SiG**, **Si-EP**, **Si-o-OR** and **Si-p-OR** are shown in Figure 1. For **SiEp**, the stretching vibration at 798 cm^−1^ and 1097 cm^−1^ correspond to **Si-O-Si**, and the weak band at 2883 cm^−1^ is due to the CH bond. In the spectra of **Si-o-OR** and **Si-p-OR**, a new peak appeared at 1460 cm^−1^ and 1463 cm^−1^, which was attributed to the characteristic C=N bending vibration. These results indicate that these materials were modified by Schiff base functional groups.

**Figure 2:**
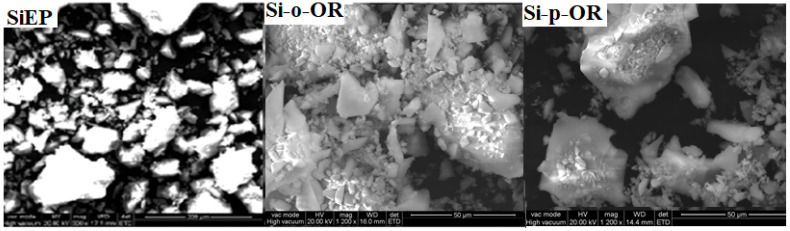
SEM images of **Si-EP**, **Si-o-OR** and **Si-p-OR** materials.

The surface morphologies of **Si-o-OR** and **Si-p-OR** were observed by scanning electron micrographs (SEM). As displayed in Figure 2, both materials present similar rough surfaces, with a wide distribution of particle micrometer-measured size. In contrast, both materials were better dispersed than **SiEP**. The results confirm the successful introduction of Schiff base ligands over **SiEP**.

**Figure 3:**
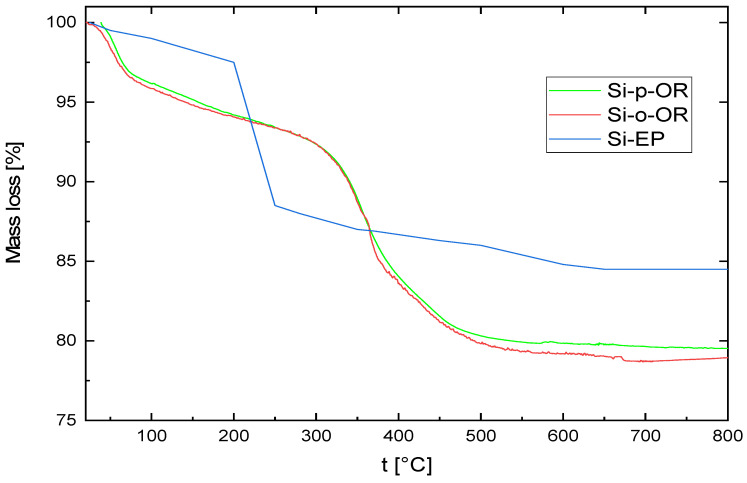
Thermogravimetric profiles of **Si-EP**, **Si-o-OR** and **Si-p-OR**.

Thermogravimetric analyses of **SiEP, Si-o-OR** and **Si-p-OR** were undertaken (Figure 3). **SiEP** show a mass loss of 10.8% between 200 and 800 °C, which corresponds to the decomposition of the silanol groups bound to the surface. For **Si-o-OR** and **Si-p-OR**, two decomposition steps are observed. The first one presents a weight loss of 3.98% for **Si-p-OR** and 4.25% for **Si-o-OR**, which starts in the range of 25–100 °C and which mainly corresponds to water loss. The second step shows a mass loss of 12.91% and 13.42% for the two hybrid materials, respectively, which can be ascribed to the decomposition of the (furan-2-ylmethyleneamino) phenol ligand.

**Figure 4:**
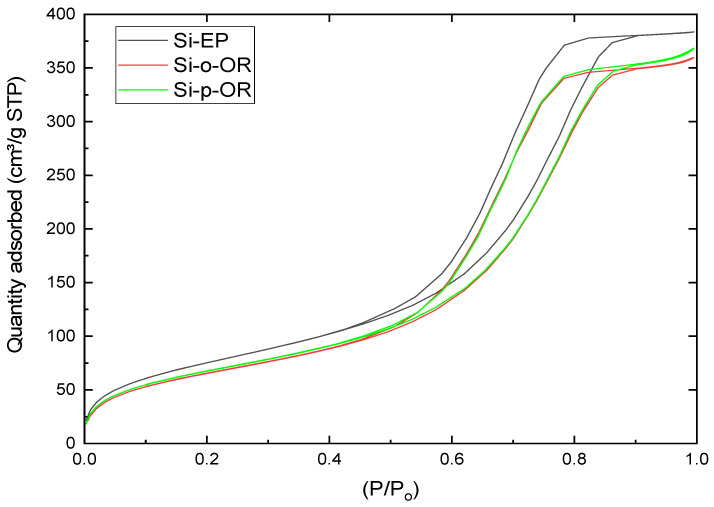
Nitrogen adsorption−desorption isotherm plots of **Si-Ep**, **Si-o-OR** and **Si-p-OR**.

The N_2_ adsorption-desorption isotherms and the corresponding BJH pore size distribution of **SiEP**, **Si-o-OR** and **Si-p-OR** were investigated. Figure 4 shows a type IV isotherm with a visible hysteresis loop, indicating an H2-type profile (IUPAC) and a uniform pore diameter distribution in the mesoporous region [29]. The BET surface area, total pore volume and pore size are 245.06 m^2^ g^−1^, 0.65 cm^3^ g^−1^ and 62.75 Å, for **Si-o-OR** and 252.12 m^2^ g^−1^, 0.66 cm^3^ g^−1^ and 64.09 Å, for **Si-p-OR**, respectively. This is as compared to **SiEP**, which demonstrates a pronounced decrease in BET surface area (277.08 m^2^ g^−1^), pore volume (0.68 cm^3^ g^−1^) and pore size (79.6 Å). These results suggest that the large surface of products was efficiently imprinted.

### 2.3. Adsorption Studies

#### 2.3.1. Effect of pH on the Adsorption of Cu(II)

**Figure 5:**
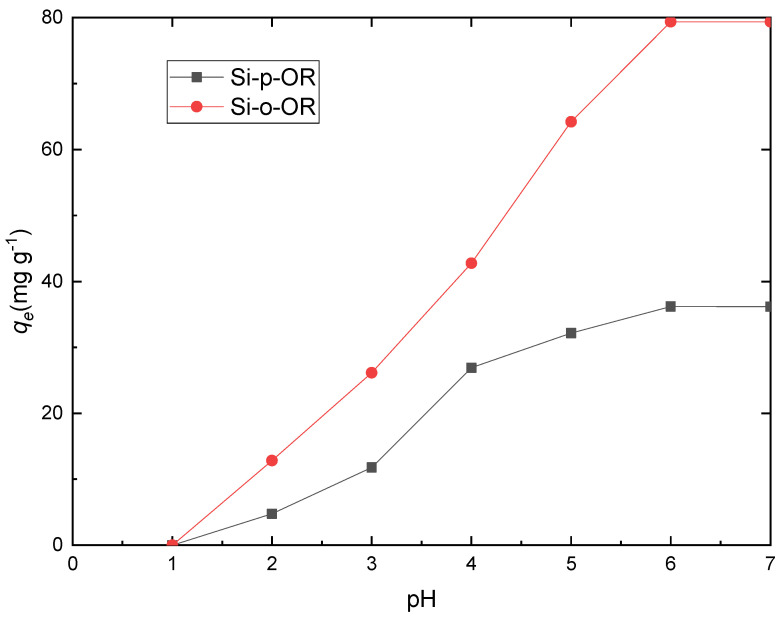
The effect of pH on the adsorption capacity of **Si-o-OR** and **Si-p-OR** towards Cu(II). Adsorption condition: V = 10 mL, m = 10 mg of adsorbent and optimum concentration = 120 mg L^−1^ for 25 min at 25 °C.

pH is a key factor to consider during the adsorption process. The influence of pH ranging from 1–7 on the adsorption of Cu(II) in both adsorbents is illustrated in Figure 5, which demonstrates that the uptake capacity of Cu(II) increased to its maximum value at pH = 6. This behaviour can be explained by the surface charge of the adsorbents with varying pH in an aqueous solution. The Schiff base and oxygen-containing functional groups on the surface of the materials are responsible for strong interactions with Cu(II) and have a high adsorption capacity on Cu(II). Comparing the results of **Si-o-OR** alone with **Si-p-OR** at different pH values demonstrates a high increase in the removal of Cu(II) (79.36 and 36.20 mg g^−1^, respectively), which suggests that the availability of the functional groups of **Si-o-OR** is higher than for **Si-p-OR**. **Si-o-OR** therefore features a high coordinating capacity for Cu(II).

At pH < 2, the adsorption capacity is low. Since the active groups of both **Si-o-OR** and **Si-p-OR** are easily protonated, N and O atoms’ electron-donating ability is weakened. At pH > 7, the abilities of **Si-o-OR** and **Si-p-OR** decrease due to Cu(OH)_2_ and CuOH^+^ formation. Overall, pH = 6 was chosen as the best condition for Cu(II) in subsequent experiments. Thus, Schiff base and oxygen-containing functional groups are good chelating agents that allow more copper ions to be adsorbed.

**Figure 6:**
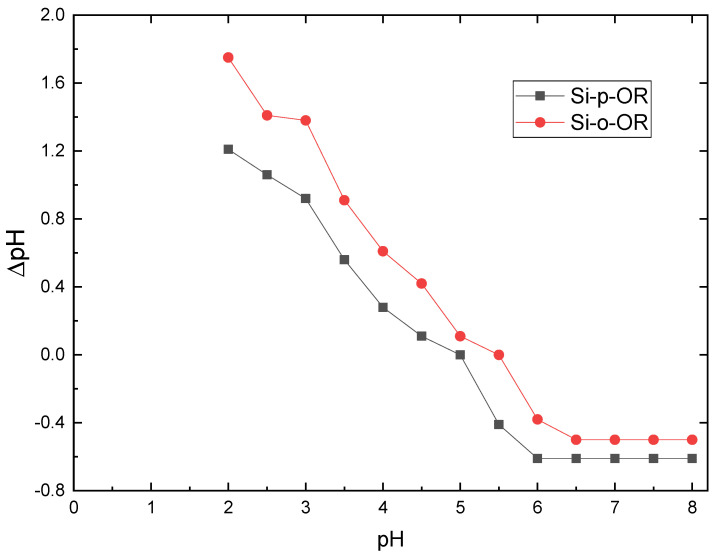
pH_PZC_ determination of **Si-o-OR** and **Si-p-OR** at different pH values. Adsorption condition: V = 10 mL, m = 10 mg of adsorbent and optimum concentration = 120 mg L^−1^ for 25 min at 25 °C.

In addition to the pH value, the point of zero charges (pH_PZC_) is one of the key parameters in studying the surface charge of the adsorbents. The pH_PZC_ of adsorbents was determined by solid addition as described in the literature [30]. Briefly, 0.01 g of **Si-o-OR** and **Si-p-OR** were mixed with aliquots of 30 mL of NaOH (0.1 M), which were prepared at different a range of different pH values (2–8) by using HCl (0.1 M) and NaOH (0.1 M) to fine-tune the pH of the solution. The point at which ΔpH = 0 (ΔpH = pH_final_ − pH_initial_) indicates pH_PZC_ of both adsorbents. The pH_PZC_ of **Si-o-OR** and **Si-p-OR** were found to be at 5.5 and 5, respectively (Figure 6). At pH < pH_PZC_, the N and O donor atoms remain in their protonated form, which results in the adsorbents bearing a positive surface charge. Higher pH values compared to pH_PZC_ indicates that the surface of the adsorbent becomes negatively charged, corresponding to deprotonation of the donor atoms.

#### 2.3.2. Effect of Contact Time and Kinetic Adsorption Modelling

**Figure 7:**
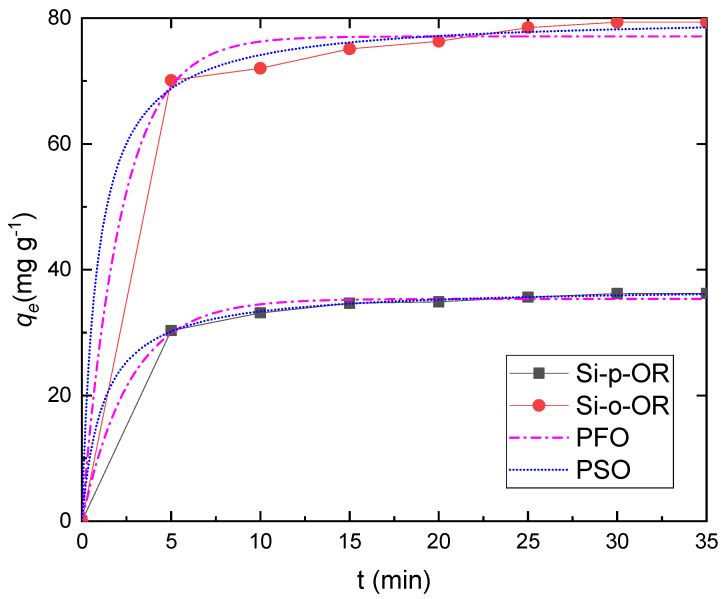
Adsorption of Cu(II) by **Si-o-OR** and **Si-p-OR** with time, fitted by a pseudo-second-order model. Adsorption conditions: V = 10 mL, m = 10 mg of adsorbent, pH = 6, and optimum concentration = 120 mg L^−1^ at 25 °C.

Contact time is known to have a great influence on the adsorption efficiency of heavy metals in aqueous solutions. Adsorption time was investigated over the range of 5–35 min. The contact effect on the adsorption behaviours of **Si-o-OR** and **Si-p-OR** beads is shown in Figure 7. The removal of Cu(II) is rapid due to a large number of donor atoms which rapidly react with copper when the adsorbent is initially exposed to the copper solution. Evidently, over 95% adsorption occurred within 15 min, and equilibrium was reached after 25 min. Subsequently, no significant change occurred with time up to 40 min, since numerous active sites on the surface of the adsorbents were consumed. Therefore, in further experiments, 25 min was selected to ensure the saturation of Cu(II) ions.

Pseudo-first-order and pseudo-second-order kinetic models were employed to analyse the adsorption kinetic data. The non-linear form of the two models can be described by Equations (4) and (5) [31,32].

Pseudo-first-order model:

q_t_ = q_e_ [1 − e^−k^_1_^t^] (1)

Pseudo-second-order model:

q_t_ = k^2^q_e_^2^t/1 + k_2_q_2_t (2)

where q_e_ (mg/g) and q_t_ (mg/g) are the amount of Cu(II) adsorbed at equilibrium and instantaneous time t respectively; k_1_ (min^−1^) and k_2_ (g/mg min^−1^) are the rate constants of the pseudo-first-order and pseudo-second-order expressions, respectively.

Fit results are gathered in Table 1 along with kinetic constants and correlation coefficients (R^2^). The R^2^ values of the pseudo-second-order model were found to be higher than the values of the pseudo-first-order model. The theoretical value q_e_ from the pseudo-second-order model best fitted the experimental values, which suggests that such a model is recommended to describe the adsorption kinetics of Cu(II) ion for both adsorbents. Therefore, chemisorption is the main factor in the rate of adsorption and involves complexation sharing between the adsorbent and the metal ions.

#### 2.3.3. Influence of Initial Concentration and Adsorption Isotherms for Cu(II)

**Figure 8:**
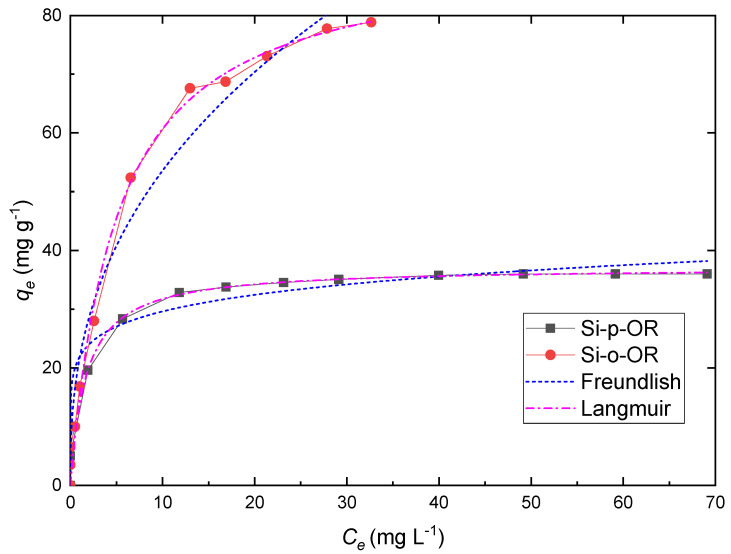
Effect of initial concentration on copper ion adsorption onto **Si-o-OR** and **Si-p-OR** and adsorption isotherms of both adsorbents towards Cu(II), fitted according to Langmuir and Freundlich models. Adsorption conditions: 10 mg, V = 10 mL, [Cu(II)] = 10 to 300 mg L^−1^, pH = 6, time = 25 min at 25 °C.

The initial concentration is one of the most important factors that provide necessary information about adsorbing performance in different concentrations. Accordingly, different initial concentrations of Cu(II) ranging from 10 to 300 mg L^−1^ were used to study the adsorbents further using the batch method. The effect of the initial concentration on the adsorption capacity of Cu(II) is illustrated in Figure 8. It can be seen that the removal of Cu(II) ion increases with an increase in Cu(II) ion concentration. This is attributed to the higher driving force arising from the high concentration gradient. The adsorption curve tends to reach a plateau corresponding to the saturated sorption monolayer.

Fitting adsorption isotherms allows for the understanding of the interaction between adsorbate and adsorbent in the uptake process [33]. The most widely applied isotherm models are Langmuir and Freundlich. The nonlinear forms of Langmuir and Freundlich models can be obtained from the following Equations (6) and (7) [34,35].

Langmuir model:

q_e_ = qK_L_C_e_/1 + k_L_C_e_ (3)

Freundlich model:

q_e_ = K_F_ · C_e_^1/n^ (4)

where q_e_ (mg g^−1^) and q (mg g^−1^) represent the equilibrium and the saturated adsorption capacity respectively; K_L_ (L mg^−1^) represents the Langmuir constant associated with the affinity of the adsorbent to the adsorbent; K_F_ (mg L^−1^) and *n* are the Freundlich constants; and C_e_ (mg L^−1^) is the equilibrium concentration of metal ions.

The nonlinear fitting curves are illustrated in Figure 8, and the characteristic parameters of these models are shown in Table 2. As a result, the correlation coefficients of the Langmuir model (R^2^) were found to fit best. Moreover, the q_e_ of both adsorbents evaluated by the Langmuir model is in agreement with the experimental value. These results demonstrate that the adsorption process is consistent with the Langmuir model of uniform monolayer sorption behaviour.

The determination of q_e_ for Cu(II) with the adsorbents **Si-o-OR** and **Si-p-OR** led to 79.36 mg g^−1^ and 36.20 mg g^−1^ respectively. Thus, the adsorption capacity of **Si-o-OR** is at least twice higher compared to the **Si-p-OR** adsorbent. Such a superior adsorption capacity could be explained by the ortho-position of the ligand grafted onto the silica surface, which adapts a geometry more suitable for chelating complexations as compared to the **Si-p-OR** ligand.

#### 2.3.4. Adsorption Thermodynamics

Thermodynamic studies were conducted to examine the effect of different temperatures on the sorption process. Thermodynamics parameters such as the Gibbs free energy change (ΔG° in kJ mol^−1^), the standard enthalpy of adsorption (ΔH° in kJ mol^−1^), and the standard entropy change (ΔS° in J mol^−1^ K^−1^) were evaluated using Equations (8)–(10) [36,37]:

K_d_ = C_o_ − C_e_/C_e_ (5)

Ln K_d_ = ΔS°/R − ΔH°/RT (6)

**Figure 9:**
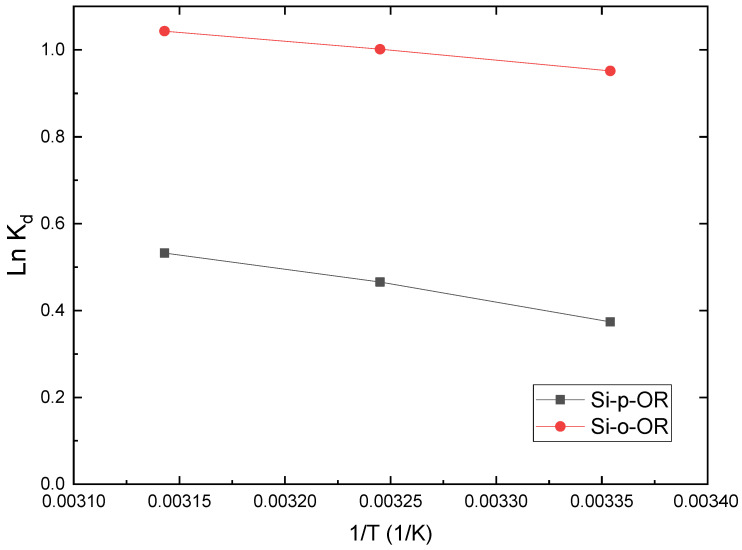
Linear plots of K_d_ *versus* 1/*T* for Cu(II) adsorption on **Si-o-OR** and **Si-p-OR**.

ΔG° = ΔH° − TΔS°(7)

where R (8.314 J mol^−1^ K^−1^) is the ideal gas constant, T is the temperature in Kelvin and K_d_ is the distribution coefficient. The numerical values of ΔH° and ΔS° could be obtained using the slope and intercept of the plot of ln (K_d_) vs. 1/*T*. The results are given in Figure 9 and Table 3. The values of ΔG° are all negative, indicating that the removal of Cu(II) on both adsorbents is a spontaneous process. Moreover, when the temperature increases, ΔG° gradually decreases, which signifies that higher temperatures favour adsorption. The positive value of ΔH° suggests the endothermic nature of the uptake process. As the values of ΔS° are also positive, this implies that the sorption is an entropy-driven phenomenon [38]. The release of water molecules from the hydration shell of adsorbed metal ions may cause the chaotic degree of the whole aqueous system and lead to an entropy increase [39].

#### 2.3.5. Selectivity of **Si-o-OR** and **Si-p-OR** Adsorbents

**Figure 10:**
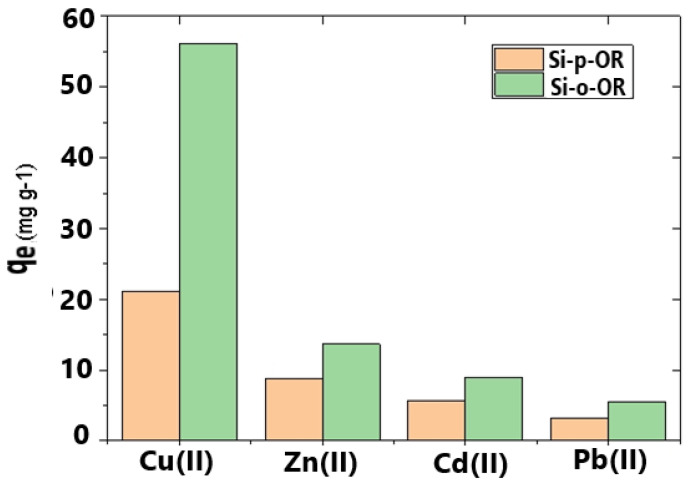
Metal ion selectivity effect using **Si-o-OR** and **Si-p-OR**.

The removal selectivity of **Si-o-OR** and **Si-p-OR** adsorbents were examined by choosing Cu(II) as representative; 10 mg of the adsorbent was added on 10 mL of mixed Cu(II), Zn(II), Cd(II), and Pb(II) quaternary systems with the same concentration (120 mg L^−1^) for competitive adsorption. As shown in Figure 10, **Si-o-OR** and **Si-p-OR** exhibit a higher adsorption selectivity for Cu(II) ion compared to other common divalent ions, such as Zn(II), Cd(II) and Pb(II). The excellent sorption selectivity of both adsorbents for Cu(II) can be explained by the ligands grafted onto the silica surface, which coordinate with Cu(II) to form more stable complexes. 

#### 2.3.6. Effect of Coexisting Ions

The presence of coexisting ions in water is another important factor influencing the adsorption and removal of Cu(II) ions. The effect of coexisting ions (K^+^, Na^+^, Ca^2+^ and Mg^2+^) was investigated when the concentration of Cu(II) ions was maintained at 0.05 μg mL^−1^. As presented in Table 4, the adsorption of Cu(II) was not weakened by any of the coexisting ions, which could not be complexed with both adsorbents.

#### 2.3.7. Desorption and Recycling

Reusability performance is an important factor for examining the extensive application of adsorbents; good recyclability can decrease costs and safeguard resources. The adsorbents **Si-o-OR** and **Si-p-OR** were evaluated in order to assess their ability to keep adsorption properties after five cycles of copper ion adsorption/desorption using HCl (2 M) as the eluent (Table 5).

After five cycles, both adsorbents retained almost 95% of their initial adsorption capacity for Cu(II) ions, which indicates that the HCl solution is efficient for the regeneration of Cu(II) loaded onto **Si-o-OR** and **Si-p-OR**. These adsorbents have good regeneration and are promising for the adsorption of Cu(II) ions from waste water.

#### 2.3.8. Application to Real Water Treatment

In order to check whether our adsorbent is applicable to real water samples, we selected two samples originating from two different Moroccan rivers: (i) the Ghiss river (located next to Al Hoceima), which demonstrated a pH = 7.7, total dissolved solids (TDS) = 1297 mg L^−1^ and conductivity *σ* = 1733 µS cm^−1^; and (ii) the Toussit-Boubekker river (in the Jerada-Oujda region), which demonstrated a pH = 7.1, TDS = 2031 mg L^−1^ and *σ* = 2301 µS cm^−1^. The adsorption capacities of **Si-o-OR** and **Si-p-OR** (10 mg) were studied under optimal conditions by the batch method using river water (10 mL). As can be seen in Table 6, the removal efficiency of Cu(II) ions as demonstrated by **Si-o-OR** was as high as 92% and 94% from the Ghiss and Toussit-Boubekker rivers, respectively. On the other hand, the percentage of removal demonstrated by **Si-p-OR** of 82% and 84% from the Ghiss and Toussit-Boubekker rivers, respectively, confirmed that this method had satisfactory recoveries and good accuracy. The results clearly indicate that the **Si-o-OR** material is an excellent candidate in the purification of real water samples.

#### 2.3.9. Comparison with Similar Adsorbents

A comparison of the performance of our adsorbents towards Cu(II) to literature examples reveal higher characteristics; in particular, **Si-o-OR** can be considered a promising candidate for wastewater treatment (Table 7).

### 2.4. Adsorption Mechanism

The adsorption mechanism was studied by combined FTIR, energy dispersive X-ray fluorescence (EDX) and computational methods.

#### 2.4.1. Energy Dispersive X-ray Fluorescence (EDX)

**Figure 11:**
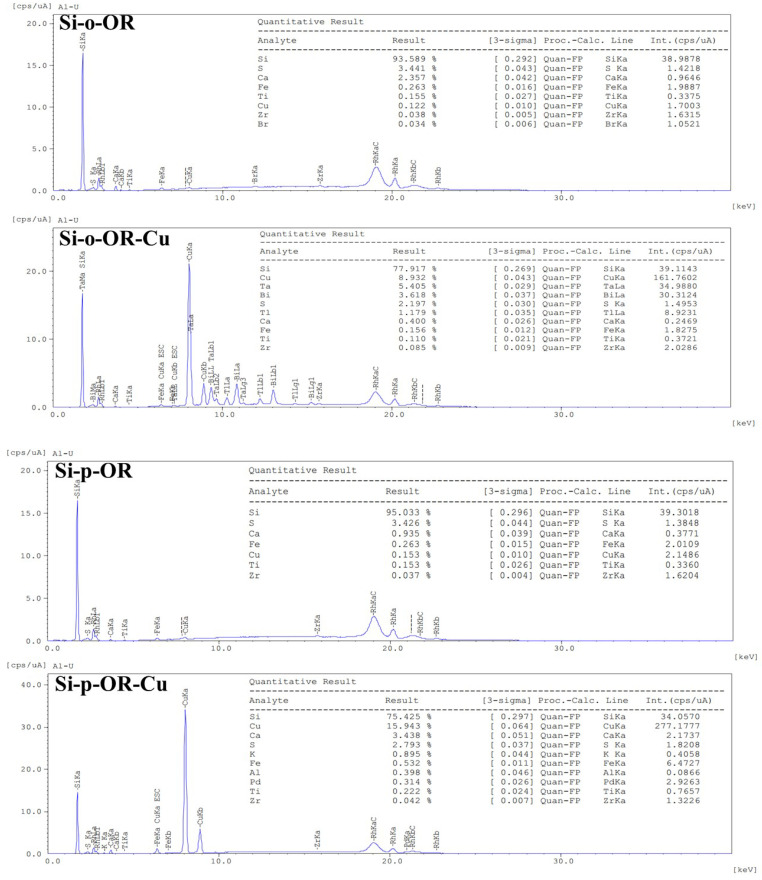
EDX spectra of **Si-o-OR** and **Si-p-OR** (before adsorption) and **Si-o-OR-Cu** and **Si-p-OR-Cu** (after adsorption of Cu(II)).

Cu(II) ions were detected after complexation as shown by comparison of EDX data of both adsorbents before and after copper ion adsorption (Figure 11). Indeed, no Cu(II) was detected in the composition of **Si-o-OR** and **Si-p-OR** before adsorption, whereas it reaches 8.93% and 15.94% for **Si-p-OR** and **Si-o-OR**, respectively.

#### 2.4.2. Fourier Transformed Infrared Spectroscopy

**Figure 12:**
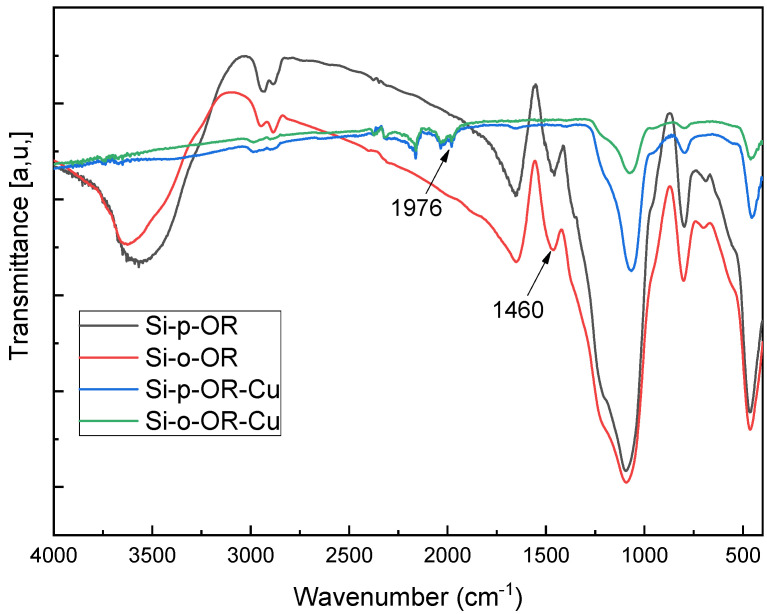
FTIR spectra before and after coordination with Cu(II) for **Si-o-OR** and **Si-p-OR**.

Figure 12 shows FTIR spectra of **Si-o-OR** and **Si-p-OR** before and after adsorption. The peak at 1460 cm^−1^ belonging to the N=C group becomes weaker after adsorption of Cu(II) ions, and is shifted downwards at 1976 cm^−1^, demonstrating the participation of N=C groups in the coordination of Cu(II) ions for both hybrid materials.

#### 2.4.3. Theoretical Investigations

##### MASD and QTAIM Calculations of Schiff Bases

**Figure 13:**
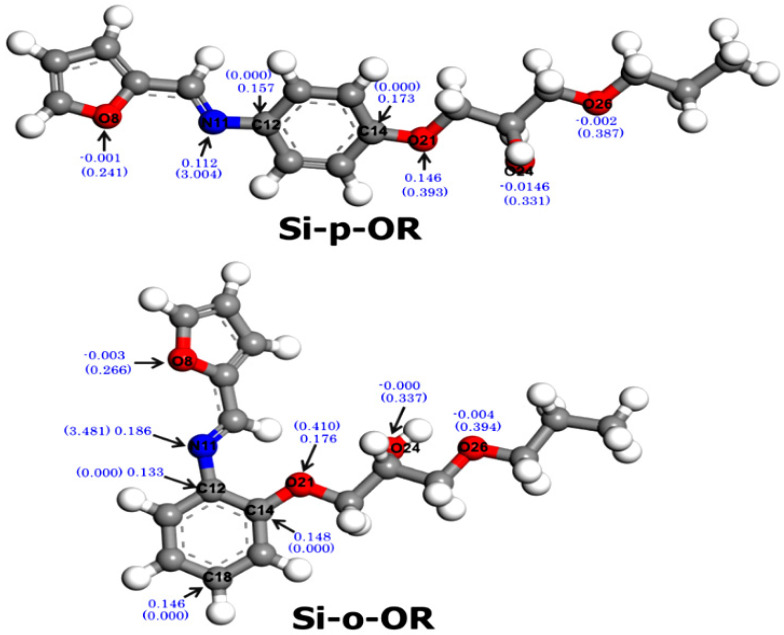
Optimized structures and *p*-values of significant atoms of Schiff bases. NBED values are given between parentheses.

Nucleophilic Parr indices (P-) are needed to evaluate the electron donating ability of atomic sites of donor species. These indices are obtained in terms of the Mulliken atomic spin density (MASD) analysis by removing an electron from the Schiff base molecule. Optimized structures of Schiff bases (**Si-p-OR** and **Si-o-OR**) and *p*-values of donor atoms are shown in Figure 13. Local reactivity was supported by QTAIM calculations of the non-bonding electron density (NBED). In particular, the NBED considers the overlap of an empty 3d orbital of Cu(II) to form chemical bonds. NBED values were therefore gathered on the same Figure 13 for completeness.

Three oxygen atoms (O8, O24 and O26) are not considered to be reactive (P- being zero or negative), which indicate that they are not involved in coordination with Cu(II). Nevertheless, a nitrogen (N11) and oxygen (O21) atom display high values of P-, indicating that these are suitable sites for an electrophilic attack via Cu(II) ions. This result was reinforced by QTAIM calculations of non-bonding electron density that aimed to describe the electron density population for each atomic space of **Si-p-OR and Si-o-OR** based on the delocalized and localized index measurements of the electron density, which is shared or exchanged between two atoms.

According to Figure 13, the most preferred active interaction sites of **Si-p-OR** and **Si-o-OR** are nitrogen atoms (N11) of the azomethine group, since their NBED values are important enough (>3 e) to establish orbital bonds with Cu(II). The situation differs for oxygen atoms (O8, O21, O26 and O28), because their NBED is not sufficient to create a novel orbital bond with the vacant d orbital (<2 e). In other words, the nitrogen atom N11 of **Si-o-OR** displays a higher value of NBED than that of **Si-p-OR**, which indicates a strong coordination of Cu(II) with **Si-o-OR**.

##### Cu(II) Complexation Study

The fully optimized geometries of possible complexes are proposed on the basis of P- indices and NBO analysis. In this sense, ΔG_C_ was calculated, which is the variation of free energy accompanying the transformation of reactant species (Cu(II) and Schiff base) to form metal complexes. Free energies of complexation (ΔG_C_) of the proposed complexes, i.e., those related to the coordination of N11-Cu(II) and Cu(II)-O21, are gathered in Table 8.

As shown in Table 8, the formation of the four proposed complexes takes place spontaneously (ΔG_C_ < 0). The comparison of ΔG_C_ values indicates that the complexes **Cu-N11(Si-p-OR)** and **Cu-N11(Si-o-OR)** present the lowest ΔG_C_ attesting the higher stability of these complexes. In addition, the ΔG_C_ corresponding to the formation of **Cu-N11(Si-o-OR)** is much lower (−112.73 kcal.mol^−1^) than that of **Cu-O21(Si-o-OR)** (−13.52 kcal.mol^−1^), which demonstrates that **Cu-N11(Si-o-OR)** is more stable; this could be explained by the fact that the **OR** group has a high electron donating character in the ortho position compared to the para position. These results indicate that **Si-o-OR** is an excellent coordinating ligand to Cu(II), which explains its high capacity to capture this metal ion from aqueous solutions as observed experimentally.

**Figure 14:**
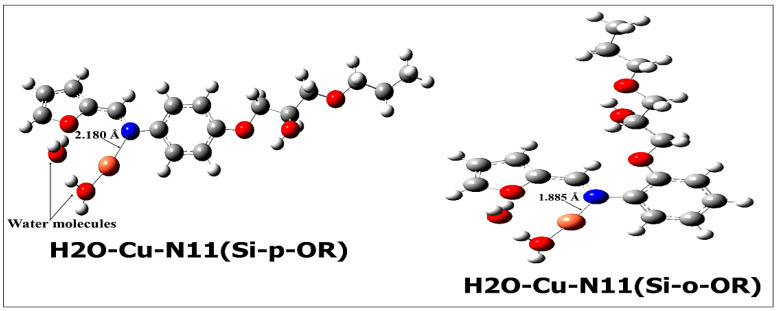
H_2_O-Cu-N11(**Si-p-OR**) and H_2_O-Cu-N11(**Si-o-OR**) complexes.

Figure 14 shows that the complexation of Cu(II) with water molecules was also considered for the complexation modes **Cu-N11(Si-p-OR)** and **Cu-N11(Si-o-OR)**. Both distance and binding energy (BE) of the length between nitrogen atoms of azomethine groups and copper atoms are also shown in Figure 14. Both **Si-p-OR** and **Si-o-OR** coordinate with Cu(II) using its nitrogen atom of azomethine groups and the oxygen atom of water molecules to form stable complexes (**H_2_O-Cu-N11-Si-p-OR** and **H_2_O-Cu-N11-Si-O-OR**); consequently, **Si-p-OR**/or **Si-o-OR** chelate with Cu(II), affording a monodentate mode. It is worthy to mention that the BE related to the N11-Cu interaction for **Si-p-OR** and **Si-o-OR** is 37.53 and 43.93 kcal/mol, respectively; this suggests that the nitrogen atom of the azomethine group coordinates strongly with Cu(II) when the OR group is in the ortho position of phenyl than in the para position.

NBO analysis allows us to study the strength of the coordination of nitrogen atoms (N11) with Cu(II) for the two studied complexes (Figure 14) with the calculation of second order stabilization energy E^(2)^ and the electronic configurations of Cu and N11 including these complexes (Table 9). Since a large value of E^(2)^ means a more intensive donor-acceptor interaction, this value is considered to be a good representation of the bond strength. According to Table 9, the valence electronic configuration of N11 in the two studied complexes ranged as follows: EC(H_2_O-Cu-N11(**Si-o-OR**) < EC(H_2_O-Cu-N11(**Si-p-OR**). For Cu, its electronic configuration varies as: EC(H_2_O-Cu-N11(**Si-p-OR**) < EC(H_2_O-Cu-N11(**Si-o-OR**). This supports the theory that there is a charge transfer process which takes place from N11 to the d-empty orbital of Cu(II), on which the coordination H_2_O-Cu-N11(**Si-o-OR**) is preferred. Otherwise, the calculated stabilization energies E^(2)^ show that the interaction strength NBED of N11→Cu(II) follows the trend: E^(2)^(H_2_O-Cu-N11(**Si-o-OR**) > E^(2)^(H_2_O-Cu-N11(**Si-p-OR**).

According to our computations, the OR group contributes its electronic density with the phenyl group because of its important mesomeric effect (+M) (an electron donating effect). In addition, the mesomeric effect (+M) of the OR group, as well as the coordination of nitrogen atoms of azomethine, is more favoured when OR is in the ortho position substituted on phenyl compared to the para position. Furthermore, the oxygen atom of the furan group contributes to its electronic density only with atoms of the furan ring.

##### NBI Analysis of H_2_O-Cu-N11(**Si-p-OR**/or **Si-o-OR**) Complexes

**Figure 15:**
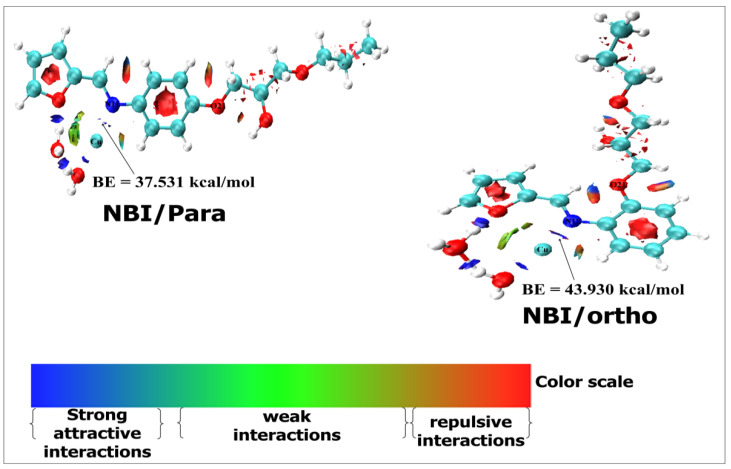
NBI analysis of two complexes: H_2_O-Cu-N11(Si-p-OR), noted NBI/para, and H_2_O-Cu-N11(Si-o-OR), noted NBI/ortho.

NBO was undertaken to characterize the nature of no-binding interactions, commonly defined as strong attractive interactions (in blue), weak interactions (in green) and repulsive interactions. Figure 15 shows the NBI shapes of the two complexes **H_2_O-Cu-N11(Si-p-OR)** and **H_2_O-Cu-N11(Si-o-OR)**; their abbreviation in this section is **NBI/para** and **NBI/ortho**, respectively.

The stronger interaction is found between the nitrogen atom (N11) of azomethine groups and copper atoms. Moreover, the oxygen atom of the furan group is connected with hydrogen atoms of the water media through a strong interaction process. This interaction is more favoured for the ortho mode of complexation: i.e., **H_2_O-Cu-N11(Si-o-OR)** than for the para mode of complexation: i.e., **H_2_O-Cu-N11(Si-p-OR)**. Furthermore, Figure 15 clearly discloses that oxygen atom (O21) coordination with Cu(II) becomes very difficult (presence of repulsive interaction). This result is in good agreement with those found in the theories based on the DFT calculations (MASD, QTAIM and NBO).

##### Adsorption Selectivity: NBO Analysis

In this section, NBO analysis was carried out to determine occupancy of d-atomic orbitals of metal ion and their energy. Table 10 shows the occupancy of d-atomic orbitals as valence orbitals (VO) and their energy for studied metal ions Cu(II), Zn(II), Cd(II) and Pb(II). Table 11 shows the energy of VO nitrogen atom N11 as the active centre of **Si-o-OR** and **Si-p-OR**.

The occupancy of d-atomic orbitals for the studied metal ions increases following the trend: Pb(II) ˂ Cu(II) ˂ Cd(II) ≈ Zn(II) (Table 10). In addition, we noticed that VO energy levels of N11 for the two studied ligands near to those of metal ions are as follows: Cu(II) ˂ Zn(II) ˂ Cd(II) ˂ Pb(II) (Table 11). Since the orbital interactions will be favourable when their energy levels are closer to each other, we can conclude that the selectivity of the ligand toward metal ions increases as follows: Cu(II) > Zn(II) > Cd(II) > Pb(II). This is in good agreement with the selectivity order observed experimentally (Figure 10).

## 3. Materials and Methods

### 3.1. Reagents and Materials

Silica gel, furfural, 4-aminophenol, 2-aminophenol, 3-glycidoxypropyl trimethoxysilane, sodium (Na), ethanol, methanol, toluene, acetic acid, tetrahydrofurane (THF), dimethylformamide (DMF), sodium hydroxide (NaOH), hydrochloric acid (HCl) and Cu(NO_3_)_2_·2H_2_O were purchased from Sigma Aldrich. Silica gel with a particle size in the range of 70–230 mesh, surface area of 470–530 m^2^ g^−1^, pore diameter of 52–73 Å, and pore volume of 0.7–0.85 cm^3^ g^−1^ was activated before use by heating at 120 °C for 24 h. The silylating agent 3-glycidoxypropyltrimethoxysilane was used without purification.

### 3.2. Experimental

#### 3.2.1. Preparation of Adsorbents

##### a-Synthesis of the Furan Derivatives

The ligands were synthesized using similar procedures to those described in previous reports [50,51]. Typically, a solution of furan-2-carbaldehyde (2 g, 20.3 mmol) and 4-aminophenol (2.3 g, 20.3 mmol) or 2-aminophenol in 20 mL of dry ethanol using 2–3 drops of acetic acid was stirred under reflux for 3 h. The resulting solids were filtered, and then crude compounds were recrystallized with hot methanol.

(E)-4-(furan-2-ylmethyleneamino) phenol, yellow powder, yield 63% (2.4 g, 12.82 mmol). m.p. 198–199 °C. R_f_ = 0.5 (silica, CH_2_Cl_2_/MeOH, 9/1). ^1^H NMR (300 MHz, DMSO) *δ* ppm: 9.52 (s, 1H, OH); 8.39 (s, 1H, CH=N); 7.85 (d, 1H, furan-Hα); 7.15 (d, 2H, phenyl, C_2_H, C_3_H); 7.03 (d, 1H, furan-Hγ); 6.77 (d, 2H, phenyl, C_3_H, C_5_H); 6.65 (m, 1H, furan-Hβ). ^13^C NMR (75 MHz, DMSO) δ ppm: 156.78 (1C, phenyl-C-OH); 152.78 (1C, C=N); 145.97 (2C, phenyl, C_2_ and C_5_) 142.75 (1C, furan-Cα); 122.86 (1C, furan-Cγ); 116.07 (2C, phenyl, C_3_ and C_5_); 112.82 (1C, furan-Cβ). *m*/*z* (M^+^): 188.21. Anal. Calcd. for C_11_H_9_NO_2_: C, 70.58; H, 4.85; N, 7.48. Found: C, 70.36; H, 4.58, N, 7.37. IR: ν(CH=N, imine) 1630 cm^−1^.

(E)-2-(furan-2-ylmethyleneamino)phenol, brown powder as 77% yield (4.5 g, 24.03 mmol). m.p. 68–69 °C. R_f_ = 0.85 (silica, CH_2_Cl_2_/MeOH, 9/1) ^1^H NMR (300 MHz, CDCl_3_) δ ppm: 8.49 (s, 1H, CH=N); 7.63 (d, 1H, furan-Hα); 7.25 (d, 1H, furan-Hγ); 7.17 (t, phenyl, C_4_H); 7.07 (s, phenyl, C_3_H); 7.01 (d, 1H, phenyl, C_6_H); 6.88 (t, phenyl, C_5_H); 5.26 (m, 1H, furan-Hβ). ^13^C NMR (75 MHz, CDCl_3_) *δ* ppm: 146.09 (1C, CH=N); 144.74 (1C, furan-Cα); 129.01 (1C, furan-Cγ); 120.10 (1C, phenyl-C_4_); 116.54 (1C, phenyl, C_3_); 115.75 (1C, phenyl-C_6_); 115.27 (1C, phenyl-C5); 110.17 (1C, furan-Cβ). *m*/*z* (M^+^): 188.04. Anal. Calcd. for C_11_H_9_NO_2_: C, 70.58; H, 4.85; N, 7.48. Found: C, 70.42; H, 4.63, N, 7, 21. IR: ν(CH=N, imine) 1605 cm^−1^.

##### b-Preparation of 3-Glycidoxypropyl-functionalized Silica **(Si-Ep)**

**Si-EP** was synthesized according to our published procedure [52,53].

##### c-Fabrication of Schiff Base-Substituted Silica: Ortho-Schiff Base Silica **(Si-o-OR)** and Para-Schiff Base Silica **(Si-p-OR)**

The hydroxy-Schiff base ligands (E)-4-(furan-2-ylmethyleneamino) phenol or (E)-2-(furan-2-ylmethyleneamino) phenol were firstly transformed into the alcoolate derivatives by applying sodium metal in THF. The suspension of 3-glycidoxypropyl-functionalized silica **Si-EP** (1 g) in 30 mL of DMF was added, and the mixture was heated to reflux under continuous stirring for 24 h. The solids **Si-o-OR** and **Si-p-OR** were filtered and washed by Soxhlet extraction with organic solvents for 12 h.

#### 3.2.2. Characterization

Elemental analyses were performed by the Microanalysis Centre Service (CNRS). FT-IR spectra were obtained using a Perkin Elmer System 2000. SEM images were obtained on a FEI-Quanta 200. Mass loss determinations were performed in a 90:10 O_2(g)_/N_2(g)_ atmosphere on a Perkin Elmer Diamond TG/DTA, at a heating rate of 10 °C min^−1^. The specific area of modified silica was determined using the BET equation. Nitrogen adsorption–desorption was obtained by means of a Thermoquest Sorpsomatic 1990 analyzer after the material had been purged in a stream of dry N_2(g)_.

#### 3.2.3. Batch Adsorption Experiments

The batch method was used in order to examine the sorption performances of Cu(II) on the modified materials **Si-o-OR** and **Si-p-OR**. For all experiments, we set the mass of adsorbent at 10 mg and the volume of the metal solution at 10 mL. Optimum parameters of pH, contact time, initial concentration and temperature were determined as follows:

- The pH value ranged from 1–7 after adjusting with dilute hydrochloric acid and sodium hydroxide solution. The uptake capacity of Cu(II) increased to a maximum value found at pH = 6;
- The effect of contact time was checked for 5–35 min. The equilibrium was reached after 25 min;
- The optimum concentration was determined by varying the initial concentration (from 10 to 300 mg L^−1^). The studies reveal that the uptake of copper ions onto both adsorbents is maximum within the following optimum conditions: m = 10 mg of adsorbent, V = 10 mL of copper ion solution, t = 25 min, pH = 6 and [Cu(II)] = 120 mg L^−1^ at 25 °C.;
- The effect of temperature was investigated over the range 25 to 45 °C.

After extraction, the solid phase was separated by filtration using a 0.45 µm nylon membrane. The residual copper concentration of the supernatant was determined by FAAS. Analyses were performed twice, and the mean data are reported. The adsorption capacity (q_e_, mg g^−1^) of adsorbents was evaluated by the following equation [54]:

q_e_ = (C_0_ − C_e_) × V/W (8)

In this formula, C_0_ (mg L^−1^) and C_e_ (mg L^−1^) refer to the initial and equilibrium Cu(II) concentrations respectively; V (mL) is the solution volume, and W (g) is the adsorbent mass.

### 3.3. Theoretical Study

The geometry optimizations of the Schiff bases and Cu complexes (**Cu@Si-o-OR** and **Cu@Si-p-OR**) were carried out with DFT(B3LYP) level of theory using the basis set 6–311G++(d,p) for H, C, N, and O atoms and LANL2DZ for the Cu atom [55,56]. Nucleophilic Parr functions (P-) of the two studied Schiff bases were obtained from the Mulliken atomic spin density (MASD). DFT, MASD and NBO calculations were performed using the GAUSSIAN 09 package [57]. Besides, NBO analysis allows the study of the role of intermolecular orbital interaction in the complex, particularly the prediction of the change in Gibbs free energies of complexation energy (ΔG_C_) and binding energy related to the length that assures coordination between the ligand and Cu(II) ion. The ΔG_C_ is calculated at 298.15 K and 1 atm by the following formula [53]:

ΔG_C_ = G_complex_ − (G_Cu(II)_ + G_Schiff base_) (9)

where G_complex_, G_Cu(II)_, and G_Schiff base_ are the free energies of the complex, the Cu(II), and the Schiff bases (**Si-p-OR** and **Si-o-OR**), respectively.

The intramolecular interactions of **Si-o-OR** and **Si-p-OR** were qualitatively evaluated in terms of NBI analysis using the multiwfn software and VMD for visualization. This approach is based on the relationship between the electron density *ρ(r)* and the reduced density gradient *s*, which is given as follows:

*s* = (1/2(3π^2^)^1/3^) · (|Δ*ρ*|/*ρ*^4/3^) (10)

This allows isosurfaces of *s* to be given at low densities, and thus allows for the visualization of the position and nature of non-binding interactions in the 3D space (either repulsive, Van der Waals, attractive or all).

Quantum theory of atoms in molecules (QTAIM) analysis using the Multiwfn software was performed in this study to obtain a greater understanding of local reactivity related to the atoms of **Si-p-OR** and **Si-o-OR** and of the kind of interaction which occurs when these ligands interact with Cu(II) (non-covalent or covalent or both) [15].

## 4. Conclusions

In summary, this study reports the preparation of two new hybrid materials, **Si-o-OR** and **Si-p-OR,** designed for the adsorption of Cu(II) ions. According to our adsorption study, the optimum pH for high uptake capacity is found at pH = 6, which favours applications under neutral conditions. The saturation of the active sites of the adsorbents is rapid and does not exceed 25 min, which is largely consistent with the pseudo-second order model that describes the adsorption kinetics of Cu(II) on the two adsorbents. Additionally, the homogeneity of the adsorption was indicated by the Langmuir model. Our thermodynamic study shows that the adsorption of copper becomes more favourable with increasing temperature, which means that the adsorption reaction is favourable. Most interestingly, **Si-o-OR** and **Si-p-OR** exhibit remarkable selectivity towards Cu(II) in the presence of different ions. Following our adsorption mechanism and computational studies, **Si-o-OR** can be considered as a promising material to remove copper ions from aquatic media.

## Figures and Tables

**Table 1:** Kinetics model data of Cu(II) adsorption.

Models	Parameters	Adsorbents	
		Si-o-OR	Si-p-OR
	q_ex__p_ (mg g^−1^)	79.36	36.20
Pseudo-first-order	q_e_ (mg g^−1^)	76.00	34.00
	k_1_ (min^−1^)	0.45	0.36
	R^2^	0.993	0.996
Pseudo-second-order	q_e_ (mg g^−1^)	80.00	36.00
	k_2_ (g mg^−1^ min^−1^)	0.01	0.02
	R^2^	0.997	0.999

**Table 2:** Parameters for the Langmuir and Freundlich models of Cu(II) sorption.

Model	Parameters	Adsorbents	
		Si-o-OR	Si-p-OR
	q_exp_ (mg g^−1^)	79.36	36.20
Langmuir	q_e_ (mg g^−1^)	88.00	36.00
	K_L_ (L mg^−1^)	0.19	0.56
	R^2^	0.99	0.98
Freundlich	N	2.50	7.50
	K_F_ (mg L^−1^)	21.00	21.00
	R^2^	0.97	0.96

**Table 3:** Thermodynamic parameters of Cu(II) using **Si-o-OR** and **Si-p-OR**.

Adsorbents	ΔH° (kJ mol^−1^)	ΔS° (J mol^−1^ K^−1^)	ΔG° (kJ mol^−1^)		
			299.15 K	309.15 K	319.15
**Si-o-OR**	3.799	20.655	−2.184	−2.586	−2.793
**Si-p-OR**	6.986	26.543	−0.955	−1.219	−1.485

**Table 4:** Effect of interfering ions on the recovery of Cu(II) ions adsorbed on both sorbents (concentration of Cu(II) ion is 0.05 μg mL^−1^).

Interfering Metal Ions	Concentration (μg mL^−1^)	Recovery of Cu(II) (%) for Si-o-OR	Recovery of Cu(II) (%) for Si-p-OR
K^+^	3000	97.89	95.98
Na^+^	3000	97. 76	94.89
Ca^2+^	2000	95. 87	92.45
Mg^2+^	2000	96.65	91.65

**Table 5:** Reusability and recycling of **Si-o-OR** and **Si-p-OR** adsorbents towards Cu(II) in repeated adsorption–desorption cycles.

Cycle	q_e_ (mg g^−1^) of Cu(II) Adsorbed on Si-o-OR	q_e_ (mg g^−1^) of Cu(II) Adsorbed on Si-p-OR
1	79.36	36.20
2	79.04	36.06
3	79.16	35.87
4	77.04	34.74
5	76.89	33.98

**Table 6:** Removal of copper from real wastewater samples using **Si-o-OR** and **Si-p-OR**.

Rivers	Added Cu(II) (mg L^−1^)	Percentage of Removal Efficiency (%) Si-o-OR	Percentage of Removal Efficiency (%) Si-p-OR
Ghiss	10	92.06	82.16
Toussit-Boubekker	10	93.87	84.65

**Table 7:** Comparison of the maximum adsorption capacities of Cu(II) by different adsorbents reported in the literature.

Silica Gel-Ligand	Reference	Metal Ion (mg g^−1^)
**Si-o-OR** **Si-p-OR**	This work This work	79.36 36.20
Porphyrin	[40]	19.08
N-propyl-2-pyridylimine	[41]	35.63
Methyl methacrylate	[42]	41.36
Dithiocarbamate	[43]	25.00
p-toluenesulfonylamide	[44]	05.00
3-Hydroxysalicylaldiminepropyl triethoxysilane	[45]	05.72
Furan ketonenol	[46]	31.82
3-amino-1,2-propanediol Commercial Lewatit (L-207) Bis(pyrazole)	[47] [48] [49]	31.18 68.09 20.24

**Table 8:** Gibbs free energies of complexation (ΔG_C_) for the studied complexes.

	ΔG_C_ (kcal mol^−1^) (Cu-N11)	ΔG_C_ (kcal mol^−1^) (Cu-O21)
**Cu-Si-p-OR**	−25.264	−05.115
**Cu-Si-o-OR**	−112.73	−13.52

**Table 9:** E^(2)^ energy calculation (kcal/mol) related to the coordination of NBED of N11 with 3d-empty orbital (3DEO) of Cu(II).

	Electronic Configuration	E^(2)^ of NBED→3DEO
	(for Cu and N11)	
**H_2_O-Cu-N11(Si-p-OR)**	**Cu:**[core]4s^0.17^3d^4.93^4p^0.06^	
	**N11:**[core]2s^1.41^2p^5.02^3p^0.01^	37.53
**H2O-Cu-N11(Si-o-OR)**	**Cu:**[core]4s^0.19^3d^7.88^4p^0.08^	
	**N11:**[core]2s^1.41^2p^5.09^3p^0.01^	43.93

**Table 10:** d-atomic orbital (d-OA) occupancies (in e) for metal ions.

d-OA	d_xy_	d_xz_	d_yz_	d_x2-y2_	d_z2_
			Occupancy		
Cu(II)	1.000	0.999	1.000	0.249	0.749
Zn(II)	2.000	2.000	2.000	2.000	2.000
Cd(II)	2.000	2.000	2.000	2.000	2.000
Pb(II)	1.999	1.999	1.999	1.999	0.000
			Energy		
Cu(II)	−0.989	−1.036	−1.092	−1.092	−1.036
Zn(II)	−1.204	−1.154	−1.204	−1.009	−1.106
Cd(II)	−1.277	−1.277	−1.277	−1.277	−1.277
Pb(II)	−1.329	−1.329	−1.329	−1.329	−1.329

**Table 11:** VO energies of N11 for **Si-o-OR** and **Si-p-OR** (in a.u.).

Ligand	2s	2p_x_	2p_y_	2p_z_
N11(Si-o-OR)	−0.337	−0.176	−0.208	−0.189
N11(Si-p-OR)	−0.503	−0.194	−0.135	−0.167

## Sample Availability

Not available.
